# Association of Sarcopenic Obesity with Higher Serum High-Sensitivity C-Reactive Protein Levels in Chinese Older Males - A Community-Based Study (Taichung Community Health Study-Elderly, TCHS-E)

**DOI:** 10.1371/journal.pone.0132908

**Published:** 2015-07-15

**Authors:** Chuan-Wei Yang, Chia-Ing Li, Tsai-Chung Li, Chiu-Shong Liu, Chih-Hsueh Lin, Wen-Yuan Lin, Cheng-Chieh Lin

**Affiliations:** 1 Ph.D. Program for Aging, College of Medicine, China Medical University, Taichung, Taiwan; 2 Department of Medical Research, China Medical University Hospital, Taichung, Taiwan; 3 School of Medicine, College of Medicine, China Medical University, Taichung, Taiwan; 4 Graduate Institute of Biostatistics, College of Public Health, China Medical University, Taichung, Taiwan; 5 Department of Healthcare Administration, College of Health Science, Asia University, Taichung, Taiwan; 6 Department of Family Medicine, China Medical University Hospital, Taichung, Taiwan; Children's National Medical Center, Washington, UNITED STATES

## Abstract

The prevalence of obesity and sarcopenia is high among the elderly. The simultaneous occurrence of these two disorders results in sarcopenic obesity. Research suggests that inflammation has an important role in the pathogenesis of obesity and sarcopenia. This study explores the impact of sarcopenic obesity on inflammatory markers, including interleukin-6 (IL-6), high-sensitivity C-reactive protein (hs-CRP), and tumor necrosis factor-alpha (TNF-α). This study is a community-based cross-sectional study. The study sample consisted of 844 community-dwelling people aged 65 years and older (448 men and 396 women). Sarcopenia was characterized by low muscle mass (skeletal muscle index < 6.87 and 5.46 kg/m^2^ for men and women, respectively), and obesity was characterized by excess body fat (body fat percentage greater than the 60th percentile of the study sample by sex [27.82% in men and 37.61% in women]). Older individuals identified with sarcopenic obesity were those who had both sarcopenia and obesity. Inflammatory markers such as IL-6, hs-CRP, and TNF-α were measured. The prevalence rates of obesity only, sarcopenia only, and sarcopenic obesity were 32.94%, 11.85%, and 7.23%, respectively. No difference was observed in the serum levels of IL-6 and TNF-α among the four groups of combined sarcopenia and obesity status. After multivariate adjustment, the serum hs-CRP levels in the obesity only and in the sarcopenic obesity groups were 0.14 and 0.16 mg/dL among males, respectively, which were significantly higher than that in the normal group (P=0.012 and 0.036). Our results provide evidence that obesity and sarcopenic obesity are associated with increased levels of serum hs-CRP among males.

## Introduction

As human beings gradually age, the basal metabolic rate and the physical activity decrease [1–3], which induce several problems such as obesity [3], metabolic syndrome [4–6], and sarcopenia (loss of fat-free mass) [3], among others. According to the Nutrition and Health Survey in Taiwan (NAHSIT), the prevalence of obesity among older people (α 65 yrs) was 18.8% from 1993 to 1996 [7]. In the 2005 to 2008 survey, the prevalence of obesity increased up to 22.0%.

Obesity is a chronic and systemic inflammatory disease, which is caused by the excessive consumption of energy that leads to the accumulation of adipose tissue. Adipose tissue secretes a number of hormones (adipocytokines) such as adiponectin, tumor necrosis factor-alpha (TNF-α), and interleukin-6 (IL-6). IL-6 stimulates the liver to synthesize acute phase proteins, which results in increased serum C-reactive protein (CRP) level. The physiological significance of CRP is to bind on phosphocholine on dead or dying cells and to clear the necrotic and apoptotic cells [8] and then induce the inflammation. Based on a previous study, high-sensitivity CRP (hs-CRP) is closely associated with abdominal obesity [9]. A few association studies have also identified the connection between hs-CRP and obesity [10, 11]. When obese patients undergo weight loss, CRP and IL-6 are significantly reduced [12]. In addition, animal experiments have provided evidence that obesity increases TNF-α levels [13], which results in chronic systemic inflammation [14].

Sarcopenia is a syndrome characterized by prominent muscle wastage among the elderly [15, 16]. The age-associated loss of muscle strength, i.e. dynapenia, is accompanied by sarcopenia [17]. Sarcopenia, low muscle mass, and dynapenia, low muscle strength, are both associated with increased risk of mobility decline in elderly. Low muscle mass is an independent predictor of mobility loss and its effect depends on muscle strength [18], indicating sarcopenia and dynapenia represent different properties and sarcopenia interacts with dynapenia on physical function. Increasing muscle mass and strength by resistance training can improve physical function in older men and women [19, 20]. Lower strength is thought to contribute to the high risk of adverse outcomes such as falls, poor quality of life, and death [21–23]. Studies have demonstrated the relationship between inflammatory markers (IL-6, CRP, and TNF-α) and muscle mass and strength among the elderly [24–26]. In the Longitudinal Aging Study of Amsterdam, high levels of IL-6 and of CRP were also associated with an increased risk of muscle strength loss [26]. According to the study by Visser et al., higher plasma concentrations of IL-6 and TNF-α were associated with lower muscle mass and muscle strength [25]. Animal experiments have also showed that IL-6 or TNF-α induces skeletal muscle protein breakdown among rats [27, 28].

Combining the two above conditions, excess body fat and loss of muscle mass result in sarcopenic obesity [29]. Sarcopenic obesity was associated with mobility disability in elderly [30, 31]. It has been reported that fat mass was more strongly associated with mobility disability in very old men and women [32] and elders with sarcopenic obesity had poor physical fitness, such as static balance, walking speed, agility and aerobic capacity [33]. But the mechanism was not completely clear. Furthermore, Atkins et al. found that sarcopenic obesity men had higher risk of mortality [34]. Studies have also established the association between obesity and sarcopenia and inflammatory markers. Using high BMI or waist circumference and low muscle strength to define sarcopenic obesity, the sarcopenic obesity was associated with elevated levels of IL-6 and CRP [35]. But recently studies used high body fat combined with low skeletal muscle mass to define sarcopenic obesity [30, 36]. Moreover, the recently data show the IL-6 has both pro- and anti-inflammatory properties [37]. This study was the first to explore the association between sarcopenic obesity, defined by high body fat combined with low skeletal muscle mass, and inflammatory markers such as IL-6, hs-CRP, and TNF-α in a community-based Chinese elderly sample.

## Materials and Methods

### Study population and sampling method

This study is a community-based cross-sectional study. The study population included all residents aged 65 years and older who were registered in June 2009 as residents of the eight administrative neighborhoods of North District, Taichung City, Taiwan. Taichung is a city located in West-Central Taiwan. Taichung City had a population of over 1 million and a population density of 6,249 per km^2^ in 2009. Taichung City comprises eight districts, which include the North District. The North District comprises 36 administrative neighborhoods. We selected elders residing in eight administrative neighborhoods around our hospital as our study sample. A total of 3,997 elderly residents in these eight administrative neighborhoods were invited to participate in the study. Through household visits, we excluded 1,247 subjects because of death, institutionalization, having moved out of the area, and errors on their registry. Among the remaining 2,750 subjects, 1,347 expressed willingness to participate in the study. The overall response rate was 49.0%. However, 503 subjects refused to undertake dual-energy X-ray absorptiometry (DXA) examination or the administration of inflammatory markers. The recruitment process flow chart was shown in S1 Fig. Therefore, 844 subjects were included in the data analysis. This study was approved by the Human Research Committee of the China Medical University Hospital. All participants submitted their written informed consent.

### Assessment of body composition

We performed DXA (Lunar DPX, General Electric) to determine the body composition of the subjects. The lean soft tissue mass and the fat mass in the arms, legs, trunk, and in the entire body were determined using a manual DXA analysis software (Lunar enCORE). Body composition was also analyzed. Equipment was calibrated using a standardized employed each day.

### Definitions of sarcopenia, obesity, and sarcopenic obesity

Skeletal muscle index (SMI) was calculated by dividing the limb muscle mass (kilograms) by the square of height (meters). Sarcopenia is characterized by low muscle mass, and is defined as having an SMI that is two standard deviations (SD) or more below the sex-specific means among young adults. With the lack of a norm for SMI among young adults in the local population, we used the reference value proposed by Sanada et al., which was derived from 529 Japanese young adults aged 18 to 40 years old [38]. The cut-off points of low muscle mass were 6.87 and 5.46 kg/m^2^ for men and women, respectively. Obesity was defined based on the reference values by Baumgartner et al. [30]. The cut-off point of obesity was a body fat percentage that is greater than the 60^th^ percentile of the study sample by sex (27.82% in men and 37.61% in women). Sarcopenic obesity is characterized by high body fat combined with low skeletal muscle mass [30].

### Measurement of inflammatory markers

hs-CRP levels were measured using a fully automatic biochemical analyzer (Unicel DxC 800 Synchron Clinical System; Beckman Coulter, Fullerton, CA, USA). The inter- and intra-assay coefficients of variations (CVs) were <2.0% and <1.9%, respectively. The lower detection limit of the assay was 0.01 mg/dL. IL-6 and TNF-α levels were measured via immunometric enzyme immunoassay (Quantikine HS, high sensitivity, R&D Systems, Minneapolis, MN, USA). The inter- and intra-assay CVs of IL-6 were <7.4% and <7.8%, whereas those of TNF-α levels were <6.7% and <13.4%, respectively. The lower detection limits of IL-6 and of TNF-α levels were 0.1 and 0.2 pg/mL. Biochemical markers, such as total cholesterol, high-density lipoprotein-cholesterol (HDL-C), low-density lipoprotein-cholesterol (LDL-C), and triglyceride, were analyzed using an automatic biochemical analyzer (Unicel DxC 800 Synchron Clinical System; Beckman Coulter, Fullerton, CA, USA) at the Clinical Laboratory Department of the China Medical University Hospital.

### Sociodemographic factors and life style behaviors

Data on sociodemographic characteristics, including age, sex, educational attainment, smoking, drinking, physical activity, physician-diagnosed diseases, fall history and medication history were collected by questionnaires. For recreational physical activity, participants who exercised for at least 30 minutes three times per week during the preceding 6 months were classified as having regular exercise. To validate the regular exercise status used in this study, we used a single question asking about with the habit of leisure time activity in health behavior questionnaire to assess their concordance rate. Regular exercise status and the habit of leisure time activity had a high concordance rate (91.0%), indicating that the regular exercise status defined in this study has concurrent validity. Moreover, the validity of this physical activity assessment tool had been evaluated in our previous study [39]. Smoking will be categorized as never, current and former. Former smokers are those who smoke at least 100 cigarettes during their lifetime but who do not currently smoke cigarettes.

### Statistical analysis

Socio-demographic factors and chronic problems of the subjects were reported as percentages or mean ± standard deviations (SD). Anthropometric measures and clinical indices were reported as mean ±SD. Differences in proportions and means were assessed using a χ^2^ test or a two-sample t-test. Post-hoc tests on the anthropometric measures and clinical indices were performed using the Tukey's method. We determined the relationship of the inflammatory markers separately with sarcopenia, obesity, and sarcopenic obesity by using linear regression models. The inflammatory markers include IL-6, TNF-α, and hs-CRP. Since the distribution of IL-6, TNF-α, and hs-CRP levels were skewed to the right, the natural log-transformation was used to normalize the data. The adjusted geometric means for hs-CRP were shown. Moreover, we used multiple linear regression models to control the possibility of confounding factors such as age, sex, cigarette smoking, exercise behavior, hypertension, arthritis, and fall history. All p values were of two-sided tests, and the level of statistical significance was set at P < 0.05. All analyses were performed using SAS version 9.1 (SAS Institute Inc., Cary, NC).

## Results

The proportions of subjects in the normal, obesity only, sarcopenia only, and in the sarcopenic obesity groups were 48.0%, 32.9%, 11.8%, and 7.2%, respectively. The prevalence of sarcopenic obesity was 7.37% among men and 7.07% among women. Distributions based on age, cigarette smoking habits, and exercise behavior were significantly different among the four groups of combined sarcopenia and obesity status (Table 1). The mean age was significantly different among the groups. Lower proportions were observed among subjects who were non-smokers and who performed regular exercises, specifically in the sarcopenic obesity group compared with those in the normal group. However, no significant difference was observed in the distributions based on sex, education, and alcohol consumption between the groups. Moreover, the distributions based on the occurrences of hypertension, hyperlipidemia, and arthritis were significantly different among the groups. The percentages of hypertension, hyperlipidemia, and arthritis in the obesity only and in the sarcopenic obesity groups were higher than those in the other groups. The percentages of fall history in the normal, obesity only, sarcopenia only, and in the sarcopenic obesity groups were 19.0, 23.2, 26.0, and 32.8, respectively, and the differences were at borderline significant (P = 0.060). Moreover, compared the statin drugs (cholesterol-lowering drugs) used between these four groups, but did not observe any significance different.

**Table 1:** Subject Characteristics by Sarcopenic Obesity Status.

	Nonsarcopenic		Obesity		Sarcopenia		Sarcopenic		
	nonobesity (n = 405)		only (n = 278)		only (n = 100)		Obesity (n = 61)		
	N	(%)	N	(%)	N	(%)	N	(%)	P value
Sociodemographic factors									
** Age (mean±SD)**	72.8 ± 5.7		74.1 ± 6.0		76.4 ± 7.0		76.5 ± 6.8		**<0.001** ^**a**^
** Gender**									0.617^b^
Men	209	(51.6)	147	(52.9)	59	(59.0)	33	(54.1)	
Women	196	(48.4)	131	(47.1)	41	(41.0)	28	(45.9)	
** Education**									0.367 ^b^
** **Illiterate	50	(12.7)	21	(7.8)	16	(16.0)	3	(5.2)	
≦6 years	102	(25.8)	74	(27.4)	27	(27.0)	17	(29.3)	
7–12 years	137	(34.7)	94	(34.8)	29	(29.0)	19	(32.8)	
≧13 years	106	(26.8)	81	(30.0)	28	(28.0)	19	(32.8)	
Health-related pratice									
** Smoking**									**0.009** ^b^
Never	332	(82.0)	219	(79.1)	68	(68.0)	47	(77.0)	
Current	36	(8.9)	19	(6.9)	16	(16.0)	3	(4.9)	
Former	37	(9.1)	39	(14.1)	16	(16.0)	11	(18.0)	
** Drinking**									0.318^b^
Never	322	(79.5)	224	(80.9)	75	(75.0)	51	(83.6)	
Current	60	(14.8)	34	(12.3)	19	(19.0)	4	(6.6)	
Former	23	(5.7)	19	(6.9)	6	(6.0)	6	(9.8)	
** Exercise**									**<0.001** ^b^
No	79	(19.7)	64	(23.0)	34	(34.3)	24	(39.3)	
Yes	322	(80.3)	214	(77.0)	65	(65.7)	37	(60.7)	
Chronic problem / Illness									
** Hypertension**									**<0.001** ^b^
No	221	(55.7)	98	(35.5)	61	(61.6)	27	(45.0)	
Yes	176	(44.3)	178	(64.5)	38	(38.4)	33	(55.0)	
** Diabetes Mellitus**									0.713^b^
No	332	(83.2)	231	(83.7)	87	(87.0)	49	(80.3)	
Yes	67	(16.8)	45	(16.3)	13	(13.0)	12	(19.7)	
** Heart disease**									0.593^b^
No	284	(71.4)	189	(69.5)	70	(70.7)	37	(62.7)	
Yes	114	(28.6)	83	(30.5)	29	(29.3)	22	(37.3)	
** Hyperlipidemia**									**0.01** ^b^
No	301	(76.2)	184	(67.9)	83	(83.8)	45	(73.8)	
Yes	94	(23.8)	87	(32.1)	16	(16.2)	16	(26.2)	
** Hyperuricemia**									0.148^b^
No	362	(90.7)	238	(86.9)	91	(91.9)	50	(83.3)	
Yes	37	(9.3)	36	(13.1)	8	(8.1)	10	(16.7)	
** Arthritis**									**<0.001** ^b^
No	322	(82.8)	186	(69.9)	85	(87.6)	44	(77.2)	
Yes	67	(17.2)	80	(30.1)	12	(12.4)	13	(22.8)	
** Stroke**									0.161^b^
No	374	(94.2)	257	(95.2)	88	(88.9)	55	(93.2)	
Yes	23	(5.8)	13	(4.8)	11	(11.1)	4	(6.8)	
** Fall history**									0.06^b^
No	325	(81.0)	212	(76.8)	74	(74.0)	41	(67.2)	
Yes	76	(19.0)	64	(23.2)	26	(26.0)	20	(32.8)	
** Statin drugs used**									0.1534^b^
No	375	(92.6)	245	(88.1)	94	(94.0)	56	(91.8)	
Yes	30	(7.4)	33	(11.9)	6	(6.0)	5	(8.2)	

^a^ Analysis by ANOVA

^b^ Analysis by Chi-square test

Furthermore, when we adjusted for the age, cigarette smoking, exercise behavior, hypertension, hyperlipidemia, arthritis, and fall history, the likelihood of having sarcopenic obesity remained significantly increasing with age (odds ratio, OR:1.08; 95% CI: 1.03 to 1.13). Exercise behavior is still significantly associated with decreased likelihood of having sarcopenic obesity (0.45; 0.24 to 0.86). After multivariate adjustment, the elderly who were older, current smokers, and who performed regular exercise were associated with higher likelihood of sarcopenia (1.09, 1.05 to 1.13; 2.19, 1.08 to 4.46; and 0.48, 0.28 to 0.81, respectively).

The anthropometric measures and the clinical indices based on subjects with combined obesity and sarcopenia status are shown in Table 2. The average weight and BMI of subjects in the groups were 59.0 ± 8.0, 68.0 ± 9.1, 50.3 ± 7.8, and 59.0 ± 7.5 kg, and 23.5±2.2, 27.3±2.9, 20.0±2.0, and 23.8±2.1 kg/m^2^, respectively, which were both significantly different (P＜0.001). The average waist and hip circumferences of subjects in the groups were 82.3 ± 7.3, 91.0 ± 8.0, 75.3 ± 7.1, and 86.1 ± 8.0 cm, and 94.9±5.0, 101.9±6.6, 90.2±5.0, and 96.5±4.9 cm, respectively, which were both significantly different (P＜0.001). The average weight and BMI of subjects in the sarcopenic obesity group were similar with those in the normal group. Moreover, the weight, BMI, and circumferences of the waist and hip in the sarcopenic obesity group were all significantly higher than those in the sarcopenia only group.

**Table 2:** Anthropometric measures and clinical indices in study subjects by sarcopenic obesity status.

	Nonsarcopenic		Obesity		Sarcopenia		Sarcopenic			
	nonobesity		only		only		Obesity			Post-hoc tests
	Mean	(SD)	Mean	(SD)	Mean	(SD)	Mean	(SD)	P value	
**Anthropometric measures**										
Weight (kg)	59.0	(8.0)	68.0	(9.1)	50.3	(7.8)	59.0	(7.5)	**<0.001**	**O > SO, N > S**
BMI (kg/m^2^)	23.5	(2.2)	27.3	(2.9)	20.0	(2.0)	23.8	(2.1)	**<0.001**	**O > SO, N > S**
Waist circumference (cm)	82.3	(7.3)	91.0	(8.0)	75.3	(7.1)	86.1	(8.0)	**<0.001**	**O > SO > N > S**
Hip circumference (cm)	94.9	(5.0)	101.9	(6.6)	90.2	(5.0)	96.5	(4.9)	**<0.001**	**O > SO, N > S**
**Clinical indices**										
Systolic blood pressure (mmHg)	137.7	(17.2)	138.9	(14.5)	137.6	(20.7)	136.1	(15.5)	0.606	
Diastolic blood pressure (mmHg)	78.3	(10.8)	79.5	(10.3)	74.5	(10.5)	75.3	(10.2)	**<0.001**	**N, O > S & O > SO**
Total cholesterol (mg/dl)	190.4	(36.9)	192.0	(31.9)	193.0	(35.9)	192.7	(38.3)	0.878	
Triglycemia (mg/dl)	116.5	(92.6)	128.5	(64.3)	95.6	(56.4)	129.4	(67.7)	**0.003**	**O, SO > S**
HDL-C (mg/dl)	47.3	(14.3)	43.4	(11.8)	50.9	(17.1)	43.0	(14.9)	**<0.001**	**N, S > O & S > SO**
LDL-C (mg/dl)	111.5	(31.0)	116.0	(28.2)	113.9	(29.0)	117.3	(32.4)	0.200	
Hs-CRP (mg/dl)	0.2	(0.4)	0.3	(0.5)	0.3	(0.7)	0.4	(0.9)	**<0.001** ^**a**^	**O, SO > N & O > S**
IL-6 (pg/ml)	4.1	(4.1)	3.9	(4.8)	4.3	(4.3)	3.8	(4.5)	0.845^**a**^	
TNF- (pg/ml)	5.1	(9.5)	4.3	(7.4)	4.0	(6.5)	3.2	(5.9)	0.553^**a**^	
			β	(SE)	β	(SE)	β	(SE)		
Ln Hs-CRP (mg/dl)^b^	baseline		**0.48** ^***^	(0.09)	0.06	(0.12)	**0.43** ^**^	(0.15)		
Ln IL-6 (pg/ml) ^b^	baseline		-0.05	(0.17)	0.11	(0.25)	-0.19	(0.30)		
Ln TNF-α (pg/ml) ^b^	baseline		0.01	(0.11)	-0.02	(0.16)	-0.27	(0.20)		

N: Nonsarcopenic nonobesity; O: Obesity only; S: Sarcopenia only; SO: Sarcopenic obesity

^a^ Analysis by natural logarithmic transformation

^b^ Using linear regression model and dependent variable being natural logarithmic transformed

^**^ P<0.01

^***^ P<0.001

For the clinical indices, the mean diastolic blood pressure, triglyceride, HDL-C, and hs-CRP were significantly different between the groups. The mean diastolic blood pressure was higher in the normal and in the obesity only groups compared with that in the sarcopenia only. The mean triglyceride was lower in the sarcopenia only group compared with that in the obesity only and sarcopenic obesity groups. The mean HDL-C in the normal and sarcopenia only groups was higher than that in the obesity only group. The mean hs-CRP increased by different sarcopenia and obesity status. In the sarcopenic obesity group, the mean hs-CRP level was the highest compared with those in the other groups.

**Fig 1:**
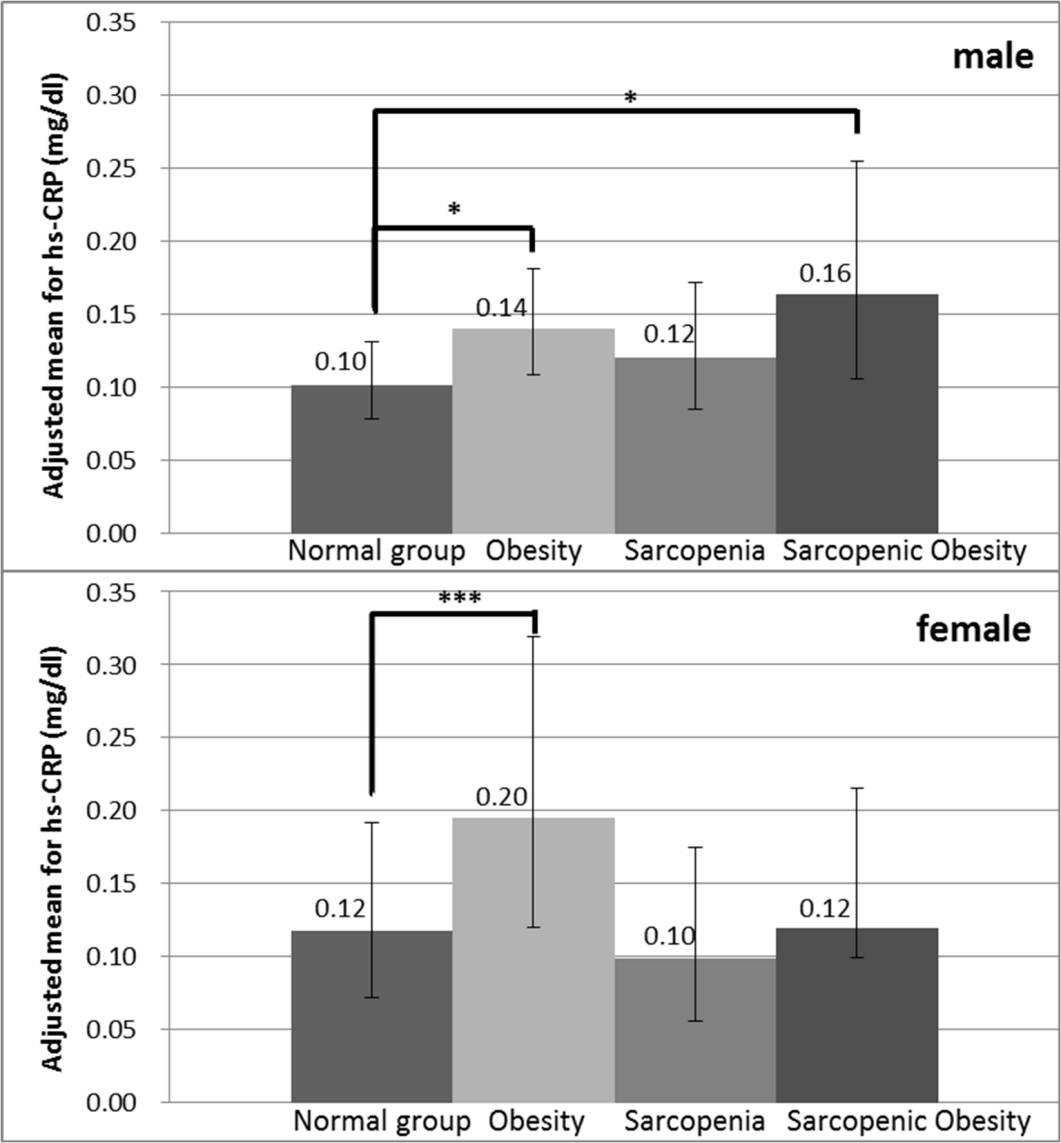
Adjusted Means for Inflammatory Marker Hs-CRP According to Obesity and Sarcopenia Status Stratified by Sex. Adjusted for age, cigarette smoking, exercise behavior, hypertension, hyperlipidemia, arthritis, fall history and statin drugs used. Data show as geometric mean. ^*^P<0.05; ^**^P<0.01; ^***^P<0.001.

Therefore, we performed regression analysis to explore the independent effects of serum hs-CRP levels in the four combined sarcopenia and obesity groups. The serum hs-CRP levels in obesity only and sarcopenic obesity groups significantly increased by 1.48-fold and 1.43-fold, respectively. No difference was observed in the serum levels of IL-6 and of TNF-α among the four groups. Moreover, we used multiple regression models to rule out the possibility of confounding factors such as age, sex, cigarette smoking, exercise behavior, hypertension, hyperlipidemia, arthritis, and fall history. The commonly prescribed cholesterol-lowering drugs, such as statins, could reduce the serum C-reactive protein levels [40]. Therefore, we also considered the statin drugs used in the multiple regression model. We further assessed the interaction between sex and sarcopenic obesity status for hs-CRP, and sex and sarcopenic obesity status had a significant interaction with each other (P = 0.016). Therefore, we separately analyzed the males and the females in the multiple regression models. Among males, the adjusted geometric means for the hs-CRP levels in the obesity only and in the sarcopenic obesity groups were 0.14 and 0.16 mg/dL, respectively (shown in Fig 1), which were significantly higher than that in the normal group. Among females, the adjusted geometric mean for the hs-CRP level in the obesity only group (0.20 mg/dL) was also significantly higher than that in the normal group. In comparisons between the other groups, we did not observe any difference. The serum levels of IL-6 and of TNF-α in the multiple regression models were not statistically different in the four groups.

## Discussion

To our knowledge, this study is the first to report on the association between sarcopenic obesity and inflammatory markers in a sample of Chinese community-dwelling elderly. Our data suggest that the serum hs-CRP levels were significantly increased by obesity and by sarcopenic obesity status. Therefore, inflammation may have an important role in the development of sarcopenic obesity. In the past, a few similar studies [35, 41, 42] have been conducted. According to a study in Finland by Stenholm et al., high body fat and low grip strength led to an increase in CRP levels [42]. Another study in Korea by Kim et al. reported that serum CRP levels were independently associated with sarcopenic obesity among women [41]. By contrast, we found that the serum hs-CRP levels among males with sarcopenic obesity increased, but not among females. In our study, no female patients with chronic hepatitis in the sarcopenic obesity group were observed, and the prevalence of chronic hepatitis among females in the sarcopenic obesity group was lower compared with that in the other groups (8.3%). A previous study revealed that chronic hepatitis increases serum hs-CRP levels [43]. Therefore, the absence of association between serum hs-CRP levels and sarcopenia and obesity groups among females in our study may be due to the low prevalence of chronic hepatitis and of unobserved confounding factors. Moreover, the previous studies show the women have more fat mass and lower muscle strength than men [44–46], and the prevalence of sarcopenic obesity in women was also higher than in men [41]. But in this study, the percentage of sarcopenic obesity among women was lower than among men. Schrager et al. also discovered that sarcopenic obesity, defined by high BMI and low muscle strength, was associated with elevated levels of IL-6, CRP, and IL-1 receptor antagonist [35], which were not observed the association between sarcopenic obesity and elevated level of IL-6 in our study. That may be due to the fact that IL-6 has both pro- and anti-inflammatory properties [37]. Moreover, the anti-inflammatory function of IL-6 was through the inhibitory effects on TNF-α [47]. Therefore, the relationship between IL-6 and TNF-α and sarcopenic obesity was weaker in this study.

In this study, sarcopenic obesity was characterized by the low skeletal muscle mass, defined by two SD or more below the sex-specific means among young adults, combined with high body fat, defined by the body fat percentage greater than the 60^th^ percentile of the study sample by sex. But waist circumference in the sarcopenic obesity group in this study was lower than the previous study [48] (95.2±11.0 cm among male, 92.2±8.9 cm among female in Korean vs. 89.6±7.5 cm among male, 81.9±6.3 cm among female in our study). That may dilute the association between sarcopenic obesity and inflammation markers. However, we still could observe the relationship between sarcopenic obesity and the serum hs-CRP levels among males.

Another interesting finding of this study is the relationship between exercise behavior and sarcopenic obesity. Our data suggest that exercise behavior was inversely related to sarcopenia and sarcopenic obesity among the elderly. The relationship between sarcopenia, sarcopenic obesity and exercise behavior may be associated with the different serum hs-CRP levels. As indicated by previous studies, exercise behavior can effectively reduce serum CRP concentration [49] among the elderly [50] or among children [51]. When the elderly are subjected to 24 weeks of exercise training, their CRP levels are effectively reduced by approximately 14.4% [50]. Therefore, exercise behavior can decrease CRP levels and inflammation, which may result in the reduced incidence of sarcopenic obesity.

This study also identified the association of sarcopenia with cigarette smoking. This result is consistent with those of previous studies [52–54]. A large cross-sectional study in Hong Kong involved community-dwelling Chinese elderly, and the results indicate that cigarette smoking was the risk factor for sarcopenia [54]. Moreover, the results from a smoking cessation study indicate that the termination of smoking habits can increase muscle mass, fat mass, and body weight [55]. Therefore, smoking cessation has the capability in sarcopenia prevention.

Several limitations are worth noting. First, the study sample was recruited from a metropolitan city in Taiwan. Our study population includes individuals from the middle to the upper middle class and those that are relatively healthy, which may have lower prevalence and less severe levels of obesity and sarcopenia. These conditions may result in limited power and weaker association between serum hs-CRP levels and sarcopenic obesity. However, our study still detected significant association of hs-CRP with obesity and sarcopenic obesity. Second, the response rate in this study was 49.0%, indicating that potential selection bias might exist. To assess this possibility, we compared the distributions of age and sex between population and sample. Similar distributions of age and sex were found (percentage differences for categories of age and sex between population and sample ranging from 1.7% to 4.9%). The non-differential distributions in age and sex, indicate this kind of selection error might be random, thus, the biased results in the effect may be toward the null, a lesser threat to validity. Third, the study was a cross-sectional study. Therefore, we were unable to examine the causal relationship of these results. Fourth, the age (75.0±7.6 years) and sex (male: 46.7%) distributions of the residents in these eight administrative neighborhoods are similar to those of the populations of both Taichung City (age: 74.1±6.9 years; male: 47.9%) and Taiwan (age: 74.3±6.9 years; male: 48.1%). Furthermore, the prevalence of ever smoking (21.0%) and regular exercise (76.0%) of participants in this study are similar to those of elderly population of Taichung City (ever smoking: 20.2%; and regular exercise:74.0%), estimated by national surveys conducted by the Health Promotion Administration, Taiwan Ministry of Health and Welfare in 2005–2009. Therefore, this study sample is representative in terms of age, sex, smoking and exercise characteristics. As for other factors, our study finding can only be generalized to those populations with similar characteristics as ours. However, there are strengths in this study. The current study was performed with a community–based sample of Taichung elderly population, which is more representative than the other sampling approach.

In this study, sarcopenia and sarcopenic obesity were associated with older age and exercise behavior. In addition, we demonstrated the significant interaction between sex and sarcopenic obesity status in serum hs-CRP levels, as evidenced by higher hs-CRP levels among males in the obesity only and in the sarcopenic obesity groups compared with those among normal males. The increase in hs-CRP among male elders with sarcopenic obesity can have clinical and health management consequences and merits further studies.

## Supporting Information

S1 Fig Recruitment Process Flow Chart. (TIF)

S1 File STROBE Statement—Checklist of Items. (PDF)
