# Correction: Association of Sarcopenic Obesity with Higher Serum High-Sensitivity C-Reactive Protein Levels in Chinese Older Males—A Community-Based Study (Taichung Community Health Study-Elderly, TCHS-E)

**DOI:** 10.1371/journal.pone.0136069

**Published:** 2015-08-13

**Authors:** Chuan-Wei Yang, Chia-Ing Li, Tsai-Chung Li, Chiu-Shong Liu, Chih-Hsueh Lin, Wen-Yuan Lin, Cheng-Chieh Lin

There are errors in the Funding section. The correct funding information is as follows: This study was supported by grants from Taiwan National Health Research Institutes (NHRI-EX100-9838PI) and Taiwan Ministry of Health and Welfare Clinical Trial and Research Center of Excellence (MOHW104-TDU-B-212-113002).
