# Supplementary figures and images for: Association of Sarcopenic Obesity with Higher Serum High-Sensitivity C-Reactive Protein Levels in Chinese Older Males - A Community-Based Study (Taichung Community Health Study-Elderly, TCHS-E)
Source: PLoS One. 2015 Jul 15;10(7):e0132908. doi: 10.1371/journal.pone.0132908 (PMC4503458; doi:10.1371/journal.pone.0132908)

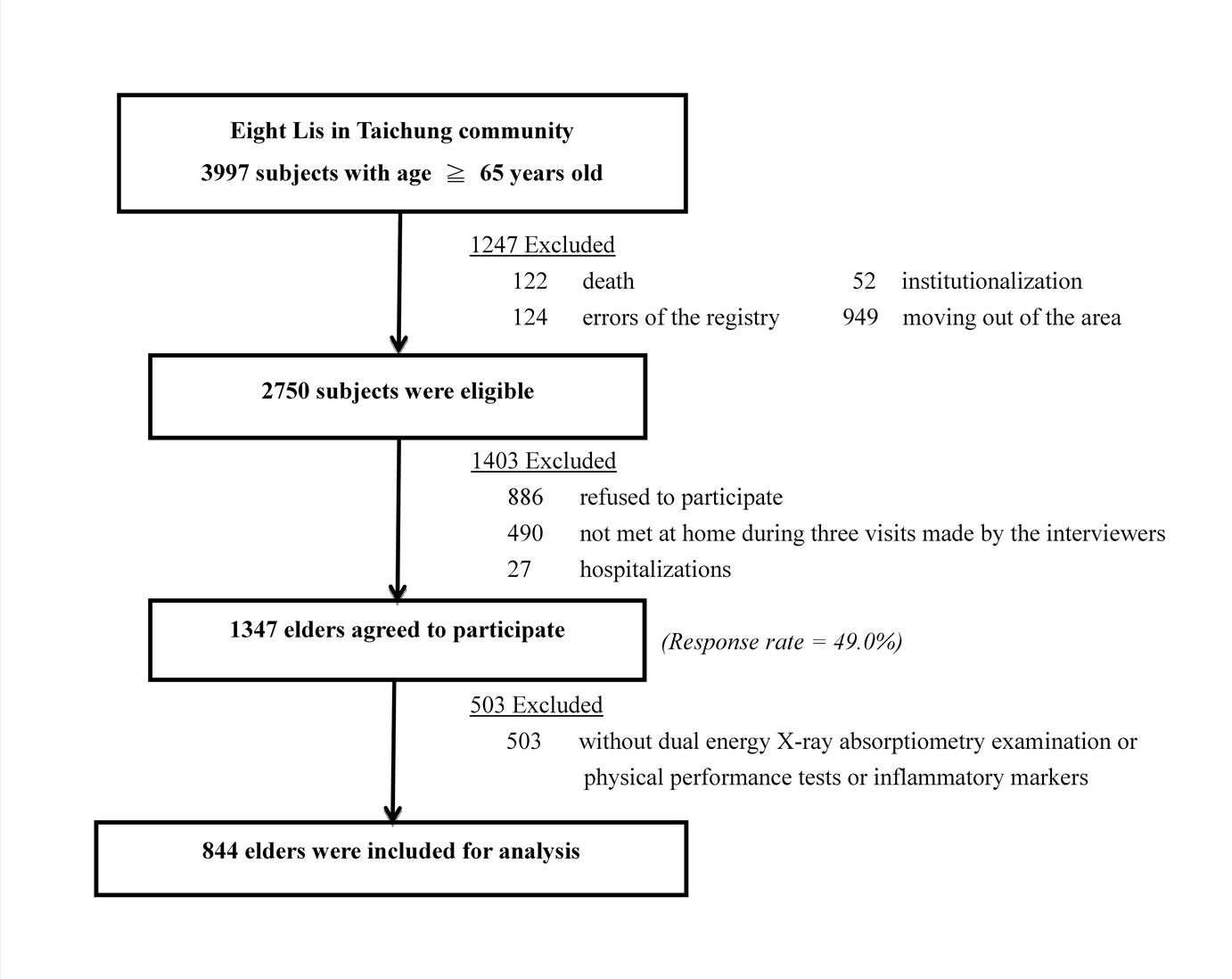

Supplement: S1 Fig — (TIF) [file pone.0132908.s001.tif]
